# A qualitative exploration of informal carers' experiences with medication management and accessing community pharmacy support for people with long-term conditions

**DOI:** 10.1016/j.rcsop.2026.100811

**Published:** 2026-06-06

**Authors:** Maha Alkhaldi, Laura Lindsey, Charlotte Lucy Richardson

**Affiliations:** a School of Pharmacy, Newcastle University, Newcastle upon Tyne NE1 7RU, United Kingdom of Great Britain and Northern Ireland; b College of Clinical Pharmacy, King Faisal University, Al Ahsa, Saudi Arabia; c Population Health Sciences Institute, Newcastle University, Newcastle upon Tyne NE1 7RU, United Kingdom of Great Britain and Northern Ireland; d Newcastle Patient Safety Research Collaborative, Newcastle upon Tyne NE1 7RU, United Kingdom of Great Britain and Northern Ireland

**Keywords:** Informal carers, Family carers, Caregivers, Medication management, Community pharmacy

## Abstract

**Background:**

People with long-term conditions often require assistance from informal carers to support medication use. Therefore, carers frequently access community pharmacies to collect medications and seek advice. This study aimed to explore the experiences and support needs of carers in medication management (MM) within community pharmacies in the United Kingdom.

**Methods:**

Carers who provided unpaid support with MM for individuals with at least one long-term condition took part in semi-structured interviews. Experiences and perspectives regarding the barriers to, and facilitators of, MM roles and accessing community pharmacies were discussed. Interviews were recorded, transcribed verbatim, and analysed using a reflexive thematic approach. Ethical approval was obtained from Newcastle University Research Ethics Committee (50254/2023).

**Results:**

Twenty-four carers were included, all of whom were familial, with most (*n* = 17) providing care for one or both parents. The sample included a wide range of perspectives, reflected by diverse MM involvement, care recipients' conditions, and carers' backgrounds. Three themes were constructed: 1) the multifaceted nature of caring, 2) carers wading through MM related information and systems, and 3) community pharmacy staff supporting carers to feel empowered in MM.

**Conclusions:**

Carers undertake a broad range of complex MM roles as part of their wider caregiving responsibilities. Improving communication between community pharmacy staff and carers is vital to support their MM role and enable safe medication use. Carer support could be tailored to each individual's unique needs. Further research is needed to address how to structure carer support from the perspective of community pharmacy staff.

## Highlights

• Carers for people with long-term conditions undertake multiple challenging medication management roles.
• Community pharmacies have the potential to support carers roles in medication management.
• Carers need meaningful conversations with community pharmacy staff to manage medications effectively and safely.
• Community pharmacy could provide carers with structured and focused support.

## 1 Introduction

Across the United Kingdom (UK), there are around 5.8 million people with caregiving responsibilities.^1^, ^2^ Carers can deliver 50 or more hours of unpaid care each week to relatives or friends,^3^ representing a vital societal contribution globally, as well as in the UK National Health Service (NHS).^3^, ^4^, ^5^ It has been noted that the number of carers in the UK is expected to rise over time as the population continues to age.^6^ An informal or unpaid carer, is ‘anyone who looks after a family member, partner or friend who needs help because of their illness, frailty, disability, a mental health problem or an addiction and cannot cope without their support. The care they give is unpaid’.^7^

Long-term conditions (LTCs) are illnesses that cannot be cured, but are controlled by medication.^8^ The prevalence of LTCs in England has steadily increased over recent decades, largely due to an ageing population.^9^ This pattern reflects a broader UK trend, with evidence indicating rising levels of LTCs across the working-age population.^10^ People living with LTCs often receive multiple medications; and taking around five or more prescribed medications is known as polypharmacy.^11^, ^12^ Polypharmacy can increase the risk of mortality and medication-related problems.^13^ Self- management of LTCs can be challenging, particularly when individuals are required to manage complex and/or polypharmacy medication regimens.^14^ Up to half of individuals with LTCs do not follow their prescriptions, causing adherence problems.^15^, ^16^ Therefore, many people rely on help from carers with their medication use.^17^

Carers play a vital role in supporting people with medication routines including medication administration.^17^ However, carers involvement in medication management (MM) raises concerns related to medication errors and safety issues.^18^ Carers often lack the training needed for safe medication administration and their education level and/or health literacy can further increase the risk of errors.^18^, ^19^ Yet many carers remain hidden and do not have support.^20^, ^21^

Community pharmacy often helps to bridge gaps during periods of high general practice (GP) demand and provides accessible health advice without the need for an appointment.^22^ For example, in England the Pharmacy First service offers walk-in support for selected conditions.^23^, ^24^ While community pharmacies have the potential to support carers, and many carers regularly visit this setting, they are still insufficiently identified and supported.^25^ Carers engagement within community pharmacy setting can be unclear and inconsistent. Even when carers identified, they receive varied levels of support from community pharmacy staff (CPS).^25^ Together, these factors highlight the growing necessity to understand how carers for people with LTCs experiences MM roles and access support within community pharmacies in the UK.

## 2 Methods

Reflexive thematic analysis according to Braun and Clarke's was used, with an inductive approach.^26^, ^27^ The consolidated criteria for reporting qualitative studies (COREQ) were used to report this work (see *Supplemental materials)*. Ethical approval was obtained from Newcastle University Research Ethics Committee (Reference: 50254/2023).

### 2.1 Research team

MA is a postgraduate researcher who has received training in conducting qualitative research. MA is an academic pharmacist with experience working in different clinical rotations across hospitals in home country only. MA conducted literature reviews as part of the PhD programme of work exploring carer roles in medication management and wider reading around community pharmacy setting in the UK. As a result of this, MA was able to develop an interpretation of the data that may have differed from the perspective a non-clinical researcher would have constructed. CR is an academic pharmacist, and LL is an academic psychologist and qualitative researcher. All members of the research team are female. No prior relationship was established with participants before the interviews.

### 2.2 Eligibility and recruitment

To be eligible participants had to be:

• Aged 18 years or above.
• Caring for an adult aged 18 years or above, who takes medication(s) for long-term condition(s).
• Has a role as an informal or familial carer (not in paid employment as a carer).
• Live in the UK.

Convenience and snowball recruitment were used to recruit potential participants. Participants were purposively selected from the pool of potential participants to ensure diversity. The study information was shared across the professional networks, social media, public involvement organisations, such as, VOICE-global.org, and carers' network and groups. A recruitment poster was made available in Arabic, Bengali, Chinese, Urdu, Punjabi, and Polish as the most common second languages locally and nationally.^28^ Written consent was obtained from the participants prior the interview.

### 2.3 Data collection and analysis

Semi-structured interviews with carers were conducted *via* Microsoft Teams, Zoom or in person in a seven-month period from October 2024 to April 2025 by MA. The interviews explored the carers’ perspectives about their MM roles, challenges, and unmet needs. Accessing relevant support from community pharmacies was also discussed. Topic guide questions were influenced by a systemic review by the research team relating to informal carers roles in MM.^17^ Each interview was transcribed verbatim, inductively coded and analysed prior to conducting the next interview. Following Braun and Clarke's reflexive thematic analysis approach, MA conducted the analysis of interview transcripts. MA repeated reading of the data to support familiarisation and generated initial, flexible descriptive codes. Codes were then developed into broader patterns of meaning, which informed the construction of themes. MA reviewed and refined these themes in an iterative process, considering how they captured the nuances of participants' perspectives. Themes were discussed with the other authors CR and LL to enhance interpretive depth, rather than seeking consensus. Themes were then reviewed, refined, named and finalised once they were coherent and analytically meaningful. An iterative constant comparison approach was used throughout the process.^29^ Sampling was guided initially by information power and conceptual depth. Subsequently reflective judgement highlighted the need to capture a broader range of lived experiences.^30^

## 3 Results

### 3.1 Participants demographics

Twenty-five participants were interviewed. One participant was excluded from the study due to ineligibility, as they were providing MM for short-term conditions only (cough, flu and infections). Three interviews were conducted in person, and 21 *via* online platforms. Interviews length varied from 27 min to 68 min (mean 44.33 min). The sample included 16 females and 8 males. The age of participants ranged from 20 to 74 years old. Participants self-reported a range of ethnicities which were subsequently mapped to census-defined ethnicity categories.^31^ The largest groups were White (*n* = 11) and Asian or Asian British (*n* = 8); followed by Black, Black British, Caribbean or African (*n* = 4), and Other (Arab; n = 1). Participants were predominantly based in England (*n* = 22), where Scotland and Wales were each represented by one participant. Most participants delivered MM support to people with multiple LTCs affecting various systems, including the endocrine, cardiovascular, respiratory, and cognitive systems. Participants reported care experiences for spouse (*n* = 6), siblings (*n* = 2), grandparents (n = 2), sister-in-law (*n* = 1) and the most common reported caring relationship was participants caring for one or both of their parents (*n* = 17). These figures represent caring relationships as some participants disclosed multiple caring experiences. Each participant was given a pseudonym based on their order of interview as (C#).

### 3.2 Thematic analysis

**Fig. 1:**
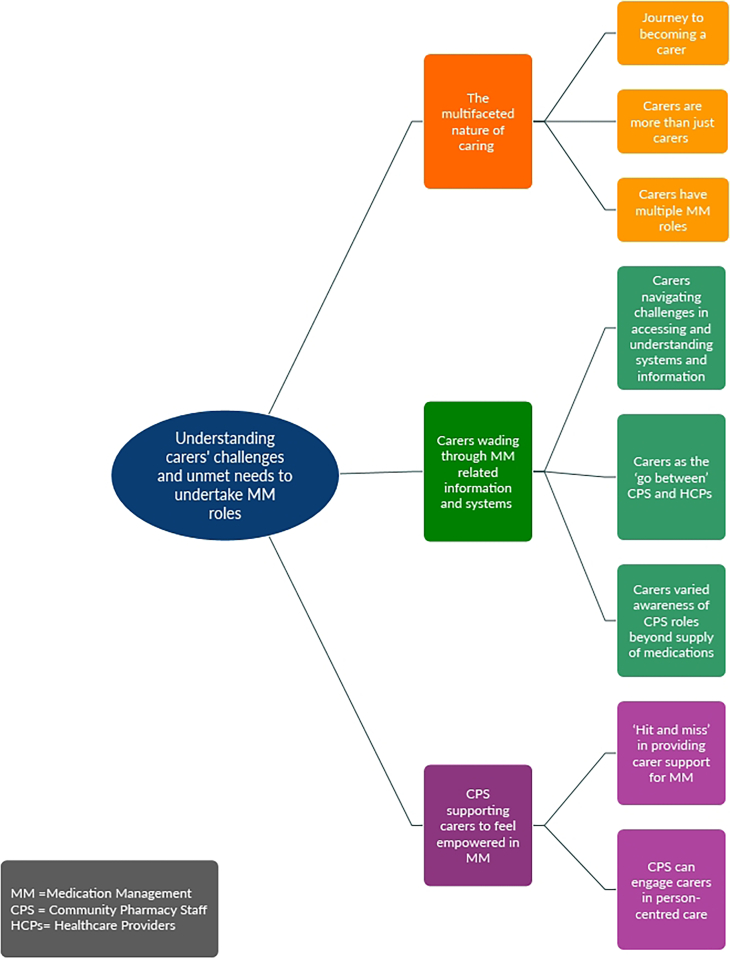
Concept map of the study themes and subthemes.

From the thematic analysis, three themes and several associated subthemes were constructed as illustrated in Fig. 1.

#### 3.2.1 Theme 1: The multifaceted nature of caring

*Journey to becoming a carer*

Participants often positioned their onset of caregiving involvement as ‘helping out’, framing their actions as valuing of family bonds and human ties rather than defining caregiving as a role. *“Charity begins at home. If you can't help your loved ones, you know, how can you help others?”* **(C5).** For example, for some participants this sense of helping evolved since childhood and was woven into family dynamics. Participants slipped into caregiving, where responsibilities evolved over time without a prior planning or decision.

*“[Being a carer] like something that you almost fall into, as I'm sure lots of people say, and that it's not like […] overnight”* **(C21).**

Participants did not describe themselves as ‘carers’ to CPS, instead they identify themselves through their family relationship for example, *“I told them, I have a brother that, he is sick, but he is an asthma patient”* **(C13).** This highlighted that participants' relational identities reinforced the view that providing care was perceived as a natural extension of being a spouse, sibling, or adult child.

*“In my head, it's just kind of normal and expected of the family… as in not that I'm pressured to do it, but it just kind of feels like a familial duty that I do. […] and like prior to this call, I don't know if I would consider myself formally with as the label of [grandmother] carer? I don't know, I might, maybe I'm not a carer!”* **(C12)**.

In addition to familial bonds, religious values, cultural norms, and personal beliefs often influenced how carers understood and defined caregiving as an expected responsibility.

*“Because it's your duty as Muslims. It's our duty to care for our parents, and obviously out of love, and sometimes because there's nobody else do”* **(C23).**

Participants' involvement in caregiving also stemmed from the challenging circumstances their care recipients faced throughout their self-management journey. For example, some participants provided care for individuals with additional needs, including hearing impairment. This often required participants to adopt further adjustments in caregiving strategies to meet specific such as sensory or language-related needs. Participants commonly acted as an advocate, spokesperson, and co-ordinator for their care recipient.

*“It's a three-way conversation, I don't just translate; I just don't translate what one says, I have to [ask questions] and explain to [the] asker”* **(C4).**

Regardless of care recipient additional needs, participants frequently acted as a care facilitator by liaising with CPS and other healthcare providers (HCPs) to bridge the gap between home and healthcare systems.

*“So, the informal care is almost the catalyst in my view [shaped a circle with his finger], that then brings in… the more professional care, the more informal care that is required”* **(C16).**  

*Carers are more than just carers*

Participants viewed caregiving for people with LTCs as burdensome, particularly as disease progression and complex polypharmacy increased the demands placed on them. This corresponded with carers' workloads and emotional strain over time.

*“So that burden is bound to increase more and so, if I talk to you in 12 months time, it might be, I might be tearing what little hair I've got out, I don't know!”* **(C16).**

Additional complexities were faced by participants who lived with their own conditions. For example, one participant described the challenges of navigating caregiving tasks while living with dyslexia. This highlighted how participants personal challenges affect caregiving responsibilities and experiences, requiring additional effort, adaptation, and emotional resilience to balance their life.

*“I have to try and read the labels, because I'm also dyslexic. […] These packages and labels and information are not designed for anyone that has, like dyslexic, or learning difficulties, or anything like that”* **(C22).**

However, participants remained willing to provide care, prioritising their care recipient's health over their own. Participants' accounts showed that their emotional wellbeing was linked with the health stability or improvement of their care recipient.

*“[Wife] just had a general anaesthetic. So, I have got [to] monitor for 24 hours and it worked sort of relatively OK on the caring role but not went so good on the fact that I have had my own medical issues”* **(C20).**

While participants strived to balance their life commitments to maintain caregiving responsibilities, a couple thought to ‘give up’. Particularly if changes in participants circumstances occurred, such as starting new employment or developing an illness.

*“If [sister-in-law] lives that long and I become ill for example also, I hope I don't; then obviously I will have to give up my caring role”* **(C5).**

However, in some instances caregiving required participants to make several scarifies to cope with intensity of care. Participants commonly echoed feelings of guilt for not being able to do more for their care recipient due to work commitments.

*“It is hard, we do find it hard, and I feel guilty a lot of the time, because I don't feel I spend enough time with [father], but I have also work”* **(C19).**

Participants' stories underscored how carers often prioritise their family's needs over themselves. For example, for some participants, employment came to an end voluntarily or involuntarily: *“I had to quit my job”* **(C2)**, whereas for others, their career pathways became restricted.

*“So [caregiving is] limiting for my career because I cannot take a job role which requires a bit of travelling here and there. I cannot take a job role which is in a fixed hours”* **(C18).**

Participants expressed frustration and highlighted broader concerns about inadequate support, especially when providing care alone. This sense of being a sole carer created feelings of isolation. This underpinned the idea that carers were expected to act beyond their capacity, suggesting this expectation normalised carers' burden.

*“It's just like I'm invisible! Everybody just goes. Oh, I'm this superhuman with this magical power. I don't need to be cared for. I don't need to be supported… it's been drowning like it's really been draining”* **(C2).**

On the other hand, participants who shared caregiving responsibilities with other family members, friends or neighbours appeared to be better able to balance their life.

*“As long as like people are in the house then I can kind of balance. […] it's almost like a shift pattern”* **(C12).**

For many participants, co-ordination was understood as a central aspect of caregiving. While joint carers were widely identified as source of support and opportunity to balance carer's life aspect, one participant perceived joint carers as a source of co-ordination disruption.

*“It would be more chaotic sometimes, […] if, you know, one person has said they're going to order medicine and does not. Or you order duplicates of medicines, or you got a lot of people crowding around at once”* **(C11).**  

*Carers have multiple MM roles*

Participants framed caregiving as multiple responsibilities not limited to single or exclusive tasks. Participants perceived caregiving as a holistic role, not limited to medication tasks but involving multiple aspects of the care recipient's health and wellbeing.

*“Caring is not one aspect; it's a very holistic aspect. You have to do a whole range of things for that person”* **(C23).**

Participants outlined wide range of MM roles, informed by the diverse lived experiences articulated by each participant. For some participants, MM was a partial responsibility, while for others it was a full-time commitment woven into their daily routines.

*“…I think I'm lucky that I'm, there is not much to do, just reminding [husband] and picking up the medications, that's it”* **(C17).**

The majority of participants secured medication for their care recipients. Participants juggled across several steps to access the right medication for their care recipient, and this was conceptualised as a cyclical process that started with ordering medication and carried through to collection. For some participants, this role extended further to include checking the collected medication or the prepared compliance aids.

*“It was when I sort of later checked the next Dosette box that came in, I noticed that there was two tablets in rather than one”* **(C20).**

Therefore, participants described being vigilant across this cycle to enable safe and effective MM. Participants reported detected, prevented potential errors, and commonly engaged in discussions with CPS to seek further support relating to accessing the right medications.

*“It's basically reconfirming, phoning the pharmacist of the pharmacy […] So, if I see a change in the dosage, I'll call them up […] Because it could be life threatening if there's… you know, a mistake”* **(C23).**

Beyond prescriptions, participants understood that their care recipient sometimes required to use over-the-counter medications. Participants checked the safety of providing such one-off medications with their care recipients regimens and ensured their appropriate use.

*“I've kind of asked, you know, is there any contraindication to taking this type of cough mixture with the medications that [husband] on?”* **(C10).**

To organise medications, participants commonly used compliance aids either those prepared by pharmacies, self-fill boxes, or their own methods. However, participants considered their care recipients' preference who might not always accept or prefer to use those tools.

*“…have you seen those [name of chocolate brand] plastic containers? Like the big containers. So basically, [mother] prefer to have her medications in there”* **(C8).**

In addition to the earlier discussed roles, participants stored medication based on the nature of particular medications, such as fridge items. Participants showed an awareness of medication-misuse risks, and thus they took additional precautionary measures to store medications in separate locations in certain circumstances such as caring for someone with cognitive impairment.

*“So, the medications that [father] takes are stored in the spare room in their house away from where my dad would routinely visit. So, that there is no risk of him”* **(C19).**

A couple of participants exerted additional effort to manage excess medication and disposal *via* pharmacies. This role was understood to support the safe and appropriate use and management of medications at home.

*“I'm stockpiling the old medication, […] then after I have to arrange it to be disposed by the pharmacy"* **(C20).**

Supporting medication administration was also part of participants routines in MM. Participants administered a wide range of medication formulations, including oral and non-oral routes. However, participants experienced greater challenges to distinguish oral medication due to similarity in appearance: *“[husband] say, ‘White small one’ and there are two small ones, so which one is that?”* **(C17).** Care recipients' cognitive, psychological, and physical limitations influenced participants to offer medication administration as a way to ensure adherence and better health outcomes.

*“I make sure that she swallows each one in my presence”* **(C5).**

Although no confusion was reported across non-oral medication appearance, one participant emphasised their effort to inspect devices, packages, and solutions before using a nebuliser.

*“So, make sure the nebules package thing wasn't broken. So, like look like it wasn't cloudy. Make sure there was no leaks, making sure it was in date, before actually using the medication in the nebuliser”* **(C9).**

Many participants acted as a prompt for their care recipients to take their medications. Reminding strategies including verbal cues, calls, alarms, place and time markers such as meals. Strategies were used either to remind care recipients or participants themselves. The complexity of reminding increased when care recipients had additional needs such as hearing impairments.

*“We can't ring [father] to remind him to take medication or remind him about things. So, it is quite difficult… so we write a lot of notes. […] it is best to have his medication box in the hall because he knows he keeps his keys in the drawer in the hall”* **(C19).**

Reminding was not limited to giving prompts for medication intake; it also extended to encourage care recipients to undertake their own self-management roles such as ordering by themselves. *“I had to remind [husband] to when to re-order”* **(C17).** Therefore, several participants focused primarily on medication monitoring to co-ordinate MM.

*“I think if [brother] omitted like time to take his medicine, I will make sure… I will call him, or I will take the medicine to him”* **(C13).**

Participants went far to monitor side effects and clinical parameters associated with medications use such as, glucose and blood pressure levels. This reflected how participants made sense of the medications effectiveness and potential risks through their MM routines.

*“[The GP doctor] said [husband] needed to do a week of blood pressure testing at home, morning and evening and then submit those. So, they could see if the dosage needed adjusting. So, I helped him with that”* **(C10).**

Most participants attempted to overcome the complexity of MM by using the provided information with medications or the prepared compliance aid. Some participants utilised tracking sheets that had been created as part of their MM routines.

*“And my uncle [joint carer] is very organised. He has like an Excel spreadsheet of all the medication [grandmother] takes. What's the daily dose? When? How much stock is left? So, that is quite good because then he shares that with all of us, and we have a record of that. So, we know exactly what she takes”* **(C12)**.

Across interlinked roles related to MM, participants sometimes made decisions about medication use independently, or they sought advice from HCPs, including CPS. Participants provided some interventions in response to what they noted, detected, or sensed during their MM.

*“There were certain things like with [mother] medication, we would just say, ‘Well, I don't think you need that anymore’. And I would ask questions to the doctor, and sometimes you go, ‘Oh, we don't need to take that one anymore”’* **(C6).**  

#### 3.2.2 Theme 2: Carers wading through MM related information and systems

*Carers navigating challenges in accessing and understanding systems and information*

Participants were frustrated by situations in which HCPs positioned carer as ‘experts’. The challenging aspect for them was being considered an expert without having the expected level of knowledge or health literacy.

*“Like the incident when my mum left the hospital with the wrong medication, the respiratory nurse said, ‘Well, did you not look at the medication?’ […] I'm not a pharmacist; I don't know what that drug is and what that drug does”* **(C24).**

Another example shared by **C14** reflected the difficulty of understanding CPS discussions. This highlighted that CPS at times failed to consider providing clear communication for carers.

*“The technicians there, they kind of provide information that I'm not so clear and they just fail to explain medication changes in a very simple term for my understanding”* **(C14).**

Participants without health-related knowledge often relied on trial-and-error learning to build confidence in MM: *“who told you that I had confidence? You just have to go through it, that's the thing”* **(C8).** In contrast, participants with health-related knowledge approached MM with a sense of greater confidence. Additionally, these participants demonstrated more effective engagement with HCPs and relevant services.

*“I've worked in a community pharmacy since I was 16, and so I have a good relationship with everybody there and I can kind of go on to the computers directly and organise it for [mother] without having to go through somebody else. So, I can kind of just manage it behind the scenes without having to you know, get a pharmacist involved and I'm quite confident around medications as well”* **(C11).**

Despite some participants had health-related knowledge that supported their understanding, they still faced uncertainty and required additional support. In particular, MM challenges emerged alongside the progression of the care recipient's condition, the evolving roles of carers, and difficulties within the healthcare system. This reinforced that carers possibly faced moments of difficulty as they navigated their caregiving responsibilities, regardless of their knowledge level.

*“I would say one thing is that for someone that's obviously got health related experience; I'm very familiar with the process of medicines and how they are managed. I was really really struggling at times to understand what was going on with my dad, and so I can't imagine for one moment how someone who doesn't have that experience and keeps clued up on things”* **(C1).**

Many participants valued discussing and obtaining information from CPS rather than reading patient information leaflets (PILs) independently. Participants critiqued the accessibility and usability of the PILs due to the presentation style or language.

*“You need a magnifying glass to be able to read all that so small writing, and then the other thing is some of the language you don't understand. So, I think being able to just, to have that a conversation with pharmacist and asking advice, and they tell us that as well”* **(C4).**

Participants searched for information independently if they felt the provided details were inadequate. For example, *“but [Dosette box] don't have patient information leaflets inside there. But I've gone online”* **(C4).** Participants used a wide variety of resources to undertake MM such as search engines, videos, NHS and pharmacy websites. A couple of participants searched information in the British National Formulary. However, participants remained unable to find the necessary information:

*“So, I knew there was the British National Formulary, […] so let me go and have a look, let me just type in, is this, you know, is this vegetarian? Can you find that information? No”* **(C22).**  

*Carers as the ‘go between’ CPS and HCPs*

Participants often described playing a proactive role to act as a ‘middleman’ between multiple HCPs, chasing matters related to medications. Participants understood the issue of poor communication between the pharmacy and GP. Therefore, participants overwhelmed by being a bridge between CPS and HCPs.

*“It always seems to be you go back to the GP and you ask them to, you know, whereas I would have thought there would be some communication between the GP and the community pharmacy, but that doesn't seem to be part of the remit anymore”* **(C21).**

Participants sensed the limited information exchange between HCPs, CPS, and carers. Participants experienced moments of being excluded, particularly when changes were made to their care recipient's medications.

*“It felt, I suppose, and which I understand, you know, you have to make changes, and but I think it was quite hard as a family member, not knowing what was going on”* **(C1).**

Participants noted that pharmacies held limited data about care recipients, and thus, pharmacy staff may not be able to respond some enquires. This meant participants needed to seek support from multiple HCPs.

*“I'm not saying the community pharmacist wouldn't know that A can affect B, it's just, for example, my mum is allergic to lots of things, right? And obviously going back on a history; the GP can see what sort of things she's been allergic to. Now, that's not something a community pharmacist can see”* **(C24).**

Many participants voiced the need for connection and collaboration between HCPs including CPS to better empower carer support. *“It's too sporadic, and it's too all over the place and if it was more joined-up then care would be better”* **(C24).** Participants echoed that pharmacists could act as moderators across the health system and signpost for further assistance.

*“I think, as a pharmacist, you do have an awareness of those different a… routes around the healthcare system. So, maybe the community pharmacist is someone that can help make sense of that”* **(C1).**  

*Carers varied awareness of CPS roles beyond supply of medications*

Participants acknowledged that CPS are the experts on medication related matters: *“…they know…often know more about the medication than the GPs do”* **(C3).** This perspective was echoed across participants who had visited pharmacies for medication-related activities. However, participants commonly collect medications without using other services.

*“So apart from getting the medication from the pharmacist, we don't really use them for anything else”* **(C7).**

Similarly, participants often mentioned medication delivery services, and they felt delivery services were an active involvement of the pharmacy in their MM, which eased the pressure of their own schedules. However, participants were not able to highlight the broader contribution of pharmacy to their role as carers. Participants perceived the pharmacy function as supplier; this perspective was echoed across participants, including those with prior health-related knowledge.

*“It's hard to see from the carer perspective…it's hard to see like what role the community pharmacy is having in almost like the safe management of like my mum's care in particular, I guess it's more complicated, more changes and you know so, and sadly, it seems like it's hard to see anything other than a supply function”* **(C21).**

Some participants discussed examples of non-medication support which mapped to public health improvement such giving *“free flu vaccinations”* **(C5)** and checking health parameters *“they check […] blood glucose”* **(C2).**

One participant was influenced to attend a pharmacy appointment due to their mother's immobility to access such services. This participant attended a structured course and did wider reading about MM, *“I'd want to be a healthcare assistant at some point in my life and I just kind fell into my lap that I became a carer”* **(C9).** Thus, this participant demonstrated greater awareness which may have influenced him to utilise wider pharmacy services.

*“I actually go and see the pharmacist myself because mum's not very mobile because of her conditions. So, I actually go into the consultation room myself and they know I'm my mum's carer”* **(C9).**

A participant with no disclosed health-related knowledge showed familiarity with pharmacist role in prescribing: *“I know that there were a group of conditions which they a lot of pharmacies said that they would prescribe medication for”* **(C3).** More advance services such as the Pharmacy First was cited by another participant. This meant awareness of pharmacy roles and services are varied across participants regardless of knowledge level and participants got to know it after issues developed during their caregiving journey *e.g.,* minor ailments.

*“I suppose I think one of the things I don't know if other people are doing it. They do what's called ‘Pharmacy First’ here now in the pharmacy where you know for minor things […] they will you know, just give you some advice […] or it may have been you know I can give you some ear drops, and I can prescribe them”’* **(C4).**

Also, across participants' stories, participants seemed to not understand the varied roles of CPS. For example, *“it's not their job to teach me”* **(C8).** Participants therefore at times underestimated what CPS could do.

*“Because their job is to dispense medication. Their job is not to be a quasi-doctor”* **(C24).**

Participants' assumptions about CPS, along with being left to explore roles and services by themselves or being forced by circumstances to do so could influence how carers received and utilised support. Thus, broader awareness of what CPS can offer to support carers is warranted.

*“Don't assume everyone knows that pharmacists do this and that and can give advice about X and Y"* **(C10).**  

#### 3.2.3 Theme 3: CPS supporting carers to feel empowered in MM

*‘Hit and miss’ in providing carer support for MM*

Most participants valued the accessibility of pharmacies in contrast to other primary healthcare settings, such as GPs which require prior appointments and long waiting times. Pharmacies were seen as first point of contact for instant care. To many participants, pharmacy locations and operational hours influenced using them to overcome the limitations of other HCPs availability or carer commitments *e.g.,* working.

*“The pharmacist is about five-minute walk from my house. So, if I ever have a problem with medication, then they your first port of call because nine times out of ten they can actually help”* **(C9).**

Additionally, the pharmacy profession is widely known for its medication expertise and often sought for basic medication-related information such as *“the pharmacist explain how the medications would work”* **(C14).** Furthermore, CPS addressed issues arising from system delays between the GP and the pharmacy when processing new changes.

*“[CPS] said, ‘Oh, but actually that medication is scored, so, you just snap it or use a little knife to cut it at the score”’* **(C10).**

However, participants were frustrated by the challenges they faced when trying to reach CPS due to the busy environment of pharmacies which worsen with staff shortages. This meant pharmacies were commonly under pressure to operate phone calls and in person services and then delayed in offering timely support.

*“[Small pharmacy] phone lines are quite busy. You know, they probably only have about six or seven staff inside the pharmacy. So, you know if you get… two or three people on the phone and you know, that is just gonna backlog everything up”* **(C20).**

While CPS were commonly described as ‘friendly’ and ‘great’ providers, pharmacies were widely criticised by participants for poor proactivity to manage supply issues including out-of-stock medication and products. Participants aimed for better co-ordination and notification as such issues could disrupt MM.

*“However, when I arrived, I was informed that the medication was out of stock. […] So, I feel like the pharmacy didn't, you know, notify me in advance about the stock shortage and I feel so down about that”* **(C15).**

Participants echoed that CPS across pharmacies often shaped carer experiences and relationships with particular pharmacies: *“the people in the pharmacy I think rather the branding pharmacies”* **(C16).** At times participants were concerned about CPS attitudes and felt frustrated when support was refused**.** For example, seeking CPS support at the last minute before pharmacy closing may not be welcomed by some staff:

*“I got in just like a minute before the you know, just before the pharmacy was about to close and the… and the guy behind the counter was not happy to serve me. But the pharmacist came out, and she was ok with it. So, the pharmacist was ok”* **(C7).**

Another example, shared by **C16** related to not getting a compliance aid for their care recipient. The participant expected prompt support and felt the staff's insistence on an ‘application’ which added unnecessary delay for requests. This mismatch between pharmacy protocol and participant expectations resulted in frustration and a sense of being undervalued, emphasising how absence of conversation about alternative options or further signposting could negatively shape the service experience.

*“When I asked about the Dosette pack ‘Oh well, [father] is low priority, you would have to put in an application, it might take a month to get it sorted out’. I'm thinking… it's a plastic box!”* **(C16).**

Although participants valued the need for conversation with CPS, a couple of participants felt privacy arrangements were insufficiently addressed. Participants touched on the sensitivity of some topics to be discuss in front of others. Pharmacy structure and culture is to handle initial questions at the front desk to keep workflow could unintentionally make carer's experiences with pharmacy ‘negative’. This contrast shows how operational standard practice can clash with individuals' values and expectations for confidentiality.

*“I don't really want you to ask me this question in front of everybody else. […] ask me the permission first*” **(C22).**

The mixed nature of pharmacy culture and operation created mixed views about CPS interactions. For example, the variation in facilities and privacy arrangement in pharmacies were affected by the pharmacy type such as supermarket pharmacy.

*“I would say depends on the pharmacy, […] some pharmacies are very helpful. I mean most of them are pretty helpful. But as they sometimes don't want to be stood in the supermarket, having the private conversations, so they all need have like a private room that you could maybe go in if it was something a bit personal”* **(C6).**

Some participants echoed that the pharmacies focus on business. Thus, trust issues were developed, which for some participants made GP services preferred over community pharmacies.

*“It shouldn't be a business model. It should be a like a care model. You know, where you can go for advice knowing that the pharmacist, the information that this pharmacist is going to give you will not be something where they are going to make some money out of it necessarily"* **(C7).**

A few participants experienced conversations initiated by CPS during medication collection to offer advice around medication use or signpost for further support: *“talk to your GP about this medication; maybe you can lower the dosage a bit, this might be a bit too much especially for that age”* **(C8).** However, several participants noted that the conversation between them and CPS during pharmacy visits was missing, and thus, development of relationships were rare. This reinforced the fact that pharmacy culture affected CPS engagement and relationships with people visiting pharmacies including carers.

*“There was just like no relationship at all, really. So, I think that… I think it's almost to me… it's almost unimaginable, because nobody has ever really even started a conversation”* **(C21).**

Some participants felt that visiting the same pharmacy and meeting the same staff each time enabled ongoing support for both carer and care recipient.

*“…when a carer sticks to one pharmacy… sticks to a single pharmacy, I feel like the pharmacy understands the person better and also the staff… the members of staff of the pharmacy becomes familiar with the needs and the medication history"* **(C14).**

For example, participants who built relationships with bilingual staff sought to maintain care recipient's ownership and ease some burdens from themselves.

*“…I've had built this relationship with a pharmacy technician and so what he does is, I've introduced my mum to him. And I said to him… and because he speaks the same language as us which is Bengali. And now, if my mum runs out of any medication, and because she doesn't know how to do online and I'm busy, and if she can't get hold of me, what she does is, she calls that person up and she said, ‘I like to speak to the pharmacy technician’, and tells his name”* **(C23).**  

*CPS can engage carers in person-centred care*

All of the participants shared suggestions to improve the way CPS support carers. Participants flagged that the experiences between carers are varied, and thus the type of needed support from pharmacy is different. However, participants still expressed what they would find helpful. To provide individualised support, participants wanted greater recognition of carers' roles and to assess their needs accordingly.

*“I suppose, you know, depends really what your caring role is, and what other demands on in your time. […] it's not a one-size-fits-all, you know, it's trying to be responsive to the individual needs of the carers”* **(C10).**

Participant suggested CPS conduct a comprehensive documentation used for tracking and evaluation. For example, **C19** expressed the need to track care recipients who had not accessed the pharmacy for a considerable length of time. Participants valued the need for identifying hidden carers and providing ongoing care.

*“So, maybe it is just [pharmacists] need kind of persona, a sheet or something about this is the patient, this is where they are on this, in that stage of life, these are the meds that they are taking, these are the family who are caring for them”* **(C19).**

Across varied stories, participants identified several facilitators that could supported their MM. Participants often made suggestions based on their current practice and challenges. For example, those with high expectations from HCPs during discussions and those self-filling compliance aids desired informational sheets or checklists to support communication with HCPs or to guide self-actions: “*maybe it's like a checklist to know which medications to put in [Dosette boxes]”* **(C9).** Furthermore, some participants wanted simpler information to be provided with the medication such as labelling medications with their indications: *“just write for high blood pressure, for diabetics”* **(C17).** Also, participants wanted to receive clearer instructions from CPS when changes happen. This meant participants valued understanding information to undertake MM appropriability.

*“Clear label, clear instructions. And one other thing, […] that every so often the name of the brand of the medication has changed, colour has changed sometimes. The size of the tablet or the pill has changed, and I think that can confuse people as well”* **(C5).**

Participants recommended additional considerations for new carers to lay basic knowledge and guidance for them about MM.

*“Maybe like a…an introductory session for new carers when they take on [MM] responsibility to actually make their carer understand what medications are for, how to use them? You know, how to look after them”* **(C9).**

Also, **C6** recommended providing information packs containing useful guidance for carers. This guidance would allow carers to understand different methods of support for a safe, effective, and less burdensome MM journey.

*“Maybe to sort of at the start of looking after somebody full time that there is an information pack that says right, this is, these are the people who can help you, this is what you can do, this is what you can claim for. This is, you know and things like you know there is a pharmacy that delivers”* **(C6).**

As the experience of MM differs between carers and evolved over time, participants expressed the need for structured support including training and education. This support could be delivered through scheduled appointments at the pharmacy or *via* home visits.

*“Maybe pharmacy appointments, you know, like, I said with a GP bit somewhere where we can just go in and chat”* **(C22).**

Many participants called CPS to involve in supporting MM together, for example by providing *“like text reminders for when you're running out or close to running out”* **(C12)** and offering constant notification *“about the status of their medication requests and provide clear explanations for stock shortages or delays”* **(C15).**

Embedding technologies to support carers were also recommended by the participants. For example, *“like robots at the front of the pharmacy"* **(C11)** or using *“like a digital portal where people could send like say photos”* **(C9).** Participants attempted to address difficulties in accessing the pharmacy during out-of-hours periods or when other circumstances limited their ability to attend directly. Participants could use digital portals as space to share concerns about their care recipient's behaviours and seek advice, such as issues related to medication refusal.

To empower carer support, **C11** proposed assigning dedicated staff to support carers needs within the pharmacy. Carers need to be valued as a partner in improving health outcomes as paid carers.

*“Maybe having like I know some pharmacies have like different people that focus on either care homes or care facilities, so that kind of speed up that process so you know having like a designated dispenser or person that you could go to, might be helpful as well”* **(C11).**

Ethnic minority participants who provided care for care recipients who speak languages other than English highlighted the importance of diverse staff within pharmacies. Particularly, bilingual staff to consider the diversity of the communities. Participants believed this would reduce cultural and language-related barriers and improve service accessibility.

*“I think the pharmacy has to represent the community it serves, because there are going to be people who don't speak English, but even people who do speak English very limited, their actual knowledge and reading ability is not very good”* **(C4).**

## 4 Discussion

This study explored the experiences of carers for people with LTCs and their perspectives about community pharmacies supporting them in MM. Carers undertake interconnected MM roles and often described experiencing multiple MM challenges. Carers perceived challenges stemmed primarily from polypharmacy, supply issues, inadequate information, medications changes, and lack of communication between HCPs including CPS. Carers seem to experience role conflict, where their relational role as family member interfered with their expected responsibilities as a quasi-professional.^32^ While CPS were willing to offer support, carers recognised barriers may limit CPS' ability to provide support which often linked to pharmacy environment and culture. An initial step to support carers in MM could be to establish meaningful conversations during community pharmacy visits to identify carers and their needs.

Carers undertake MM roles to help their care recipient's use medications safely and effectively including detecting errors. Wider evidence has reported that carers play a proactive role in identifying health problems including prescription errors and side effects before HCPs.^33^, ^34^ However, the lack of carer identification within the healthcare system, including community pharmacy, can restrict carer access to support, particularly with urgent medication related queries.^35^ In our study, it appeared that carers identified themselves by their actions, familial norms, culture obligations and as a religious duty than by the label of ‘carer’. Similarly, Robinson-Barella et al. (2025) reported that carers' identification was shaped by community expectations and carer's own beliefs.^36^

Carers tended to visit community pharmacies to collect medications, with many unaware of the wider role and support CPS can offer. Many people remain unfamiliar with the broader services community pharmacies provide, which limit their use of these services.^37^ Similar to our findings, Saramunee et al. (2014) highlighted the need for better promotion and clearer communication about what pharmacies can offer.^38^ Carduff et al. (2014) suggested that promoting carer support through visible channels in primary care, such as GP websites, waiting-room posters, in the pharmacy or information shared by district nursing teams could prompt carers to recognise their role and seek support.^39^ Therefore, more engagement is needed from CPS to actively inform and encourage carers to seek support, rather than assuming carers understand how to access support.

Consistent with our findings, carers highlighted different needs as they undertake changing roles dependent on the care recipient's needs and diagnosis.^17^, ^40^ Our findings showed that CPS often made efforts to assist carers with medication-related needs where possible with mixed success. Robinson-Barella et al. (2025) noted that carers did not always receive support from HCPs.^36^ Consequently, carers were often left to manage their care recipients' medications on their own.^35^ Prior work indicated that pharmacy support was not structured and tended to be *ad hoc*, relying on carer-driven assistance.^25^, ^41^ Community pharmacies could prioritise developing carer-centred services that meet carers' needs in MM roles as part of their professional responsibilities.^42^ Earlier study suggested that providing person-centred care for both the care recipient and the carer helps ensure that each individual's unique needs, goals, and preferences are understood and supported.^43^ However, this work broadly explored carer support during hospital discharge for MM, but did not specifically focus on CPS, highlighting the need for further research in this area.

Carers broadly suggested more information and transparency in MM related information. According to Garfield et al. (2021) proactive communication between CPS, care recipients, and carers is vital, yet a gap in support and lack of communication to access medications has been highlighted.^44^ It was noted that pharmacists need to be aware of the different information needs of carers.^42^ A study by Noureldin et al. (2025), highlighted that fostering relationships with carers and understating their information needs are essential for better interaction between pharmacist and carers.^45^ In line with our findings, community pharmacies need to strengthen their support for carers by providing consistent, accurate, and tailored information.^35^ Although system-level problems require attention, improvements may be best guided by the individual circumstances of each family.^35^ A carer focused tool could facilitate carer roles in MM and utilise pharmacy expertise around medication-related needs.

### 4.1 Strength and limitations

This study included a diverse sample of carers across ages and ethnicities who assist individuals with different LTCs. Despite advertising the study poster across multiple platforms, all participants were familial carers rather than caring for friends or neighbours. While attempts were also made to recruit non-English speakers, all interviews were conducted in English, meaning perspectives from individuals with limited English proficiency may not be represented Additional studies focusing on non-familial and other minority groups of carers are required.

## 5 Conclusion

Carers have a pivotal role in managing medication for people with LTCs, yet they often lack the structured support they would like to fulfil this role effectively. While community pharmacies serve as accessible points of support and can signpost to and connect carers with other HCPs, carers struggled across varied systems, providers, and with complex medications regimens. Recognising carers as partners in MM is needed to ensure their voices help to shape carer focused care in community pharmacies toward safer medication use.

## CRediT authorship contribution statement

**Maha Alkhaldi:** Writing – review & editing, Writing – original draft, Visualization, Validation, Software, Resources, Project administration, Methodology, Investigation, Funding acquisition, Formal analysis, Data curation, Conceptualization. **Laura Lindsey:** Writing – review & editing, Writing – original draft, Visualization, Validation, Supervision, Software, Resources, Project administration, Methodology, Investigation, Formal analysis, Data curation, Conceptualization. **Charlotte Lucy Richardson:** Writing – review & editing, Writing – original draft, Visualization, Validation, Supervision, Software, Resources, Project administration, Methodology, Investigation, Formal analysis, Data curation, Conceptualization.

## Funding statement

This work was conducted as part of doctoral studies sponsored by a scholarship from King Faisal University in Saudi Arabia and the 10.13039/100012363Royal Embassy of Saudi Arabia Cultural Bureau Attaché in London and is hosted at Newcastle University. The sponsor has no role in the collection, analysis, interpretation of data or decision to submit the article for publication.

## Declaration of competing interest

No conflict of interest.

## Data Availability

Data supporting the findings of this study can be found within the main article and/or the supplemental material document or can be acquired from the authors.

## Appendix A Supplementary data

This file contains The COREQ Checklist and the Topic guide
