# Supplementary material for: A qualitative exploration of informal carers' experiences with medication management and accessing community pharmacy support for people with long-term conditions
Source: Explor Res Clin Soc Pharm. 2026 Jun 6;23:100811. doi: 10.1016/j.rcsop.2026.100811 (PMC13312071; doi:10.1016/j.rcsop.2026.100811)
Supplement: Supplementary file 1 — This file contains The COREQ Checklist and the Topic guide [file mmc1.docx]

**The Consolidated criteria for reporting qualitative studies**

| **Number** | **Item** | **Details/page** |
| --- | --- | --- |
| **DOMAIN 1: RESEARCH TEAM AND REFLEXIVITY** | | |
| **Personal Characteristics** | | |
| 1 | Interviewer/facilitator | 4 |
| 2 | Credentials | 5 |
| 3 | Occupation | 5 |
| 4 | Gender | 5 |
| 5 | Experience and training | 5 |
| **Relationship with participants** | | |
| 6 | Relationship established | 5 |
| 7 | Participant knowledge of the interviewer | MA is a PhD student who was made aware to participants at the start of the interviews and enclosed with the participant information sheet. |
| 8 | Interviewer characteristics | 5 |
| **DOMAIN 2: STUDY DESIGN** | | |
| **Theoretical framework** | | |
| 9 | Methodological orientation and Theory | 4 |
| **Participant selection** | | |
| 10 | Sampling | 5-6 |
| 11 | Method of Approach | 5-6 |
| 12 | Sample Size | 6 |
| 13 | Non-participation | n=0 |
| **Setting** | | |
| 14 | Setting of data collection | 4 |
| 15 | Presence of non-participants | None |
| 16 | Description of sample | 5-6 |
| **Data Collection** | | |
| 17 | Interview guide | 4 - supplementary material |
| 18 | Repeat interviews | None |
| 19 | Audio/visual recording | Either |
| 20 | Field notes | MA kept a reflexive diary throughout interviews to note down observations and reflections |
| 21 | Duration | 4 |
| 22 | Data saturation | 5 |
| 23 | Transcripts returned | n/a |
| **DOMAIN 3: ANALYSIS AND FINDINGS** | | |
| **Data analysis** | | |
| 24 | Number of data coders | 4-5 |
| 25 | Description of the coding tree | 7 |
| 26 | Derivation of themes | A description of the coding process is described and a coding map can be acquired from the authors |
| 27 | Software | NVIVO V13 |
| 28 | Participant checking | n/a |
| **Reporting** | | |
| 29 | Quotations presented | Quotations presented throughout 8-27 |
| 30 | Data and findings consistent | Both quotations and interpretations are presented to allow judgement of the consistency of data and findings |
| 31 | Clarity of major themes | Presented within the manuscript 8-27 |
| 32 | Clarity of minor themes | Diverse and minority cases are highlighted within the manuscript 8-27 |

**Topic guide** (The semi-structured interview questions will be based around the following areas. The questions and lines of conversation will be guided by participant answers; further questions may be asked to explore perceptions/opinions in greater detail).

| **Questions** |
| --- |
| 1. Could you please tell me a bit about the general health for your [XX]?   **Prompts:**   1. Could you please describe the medications you help your [XX] with, particularly I’m interested about the medications type such as inhaler, tablets or injections etc? 2. Can you tell me a little bit about your caregiving role?  - What motivates you to be a carer?  1. Can you tell me please if you provide unpaid care for someone else? |
| 1. Can you provide a brief overview of your routine as a carer for your [XX]’s medications?   **Prompts:**   - Would you please elaborate, how do you [ name of task(s)] medications? - What helped you feel more confident doing these tasks? - Can you describe your main focus in managing your [XX]’s medication? - What strategies do you use to manage your [XX]’s medications? / What makes your role [easier/harder]? - How do you get information about medications? - Would you mind sharing how you feel about managing your [XX]’s medications? |
| 1. Can you tell me about the most frustrating experiences of being a carer for your [XX] especially with managing their medications?   **Prompts:**   - How do you overcome these challenges? - Would you please tell me how do you balance caregiving role with your life commitment? |
| 1. Who else is involved in caring for your [XX]’s medications?   **Prompts:**   - *If yes: How* did you find the involvement of multiple people in caring for your [XX]. - *If no:* In your opinion, who else should be involved in caring for your [XX] ? |
| 1. Would you mind sharing any experiences you have had with community pharmacy as a carer?   **Prompts:**   - How do you use the pharmacy as a carer, aside from picking up prescriptions? - *If limited,* what may prevent you from seeking support from your pharmacy?  1. How did pharmacy staff respond to your participation/query?   **Prompts:**   - As a carer, can you give an example of [negative/positive] experience you have had when asking your pharmacy for assistance? - Why were the pharmacy staff [supportive / non-supportive]?  1. As a carer, how would you describe your relationship with the pharmacy staff?   **Prompts:**   - How could your interaction with the pharmacy staff be improved?  1. In your opinion, what could pharmacy staff do to better support carers?   **Prompts:**   - How could the community pharmacy serve as a primary option for carer support? - What additional support could be provided to carers? |
| 1. Can you tell me about the most positive experiences of being a carer for your [XX] especially with managing their medications? |
| 1. Just before we finish, is there anything else you would like to add, that you think I should have asked about? |

Examples of props:

- Can you tell me more about….
- What happened next…
- How did that make you feel…
- Could you expand on that…
- Could you please give an example of…….
- Would you please elaborate a little bit
